# Impact of different parameters on the outcome of vv-ECMO therapy in burn patients – a retrospective cohort study from a burn and high output ECMO center

**DOI:** 10.1515/iss-2024-0024

**Published:** 2024-09-16

**Authors:** Alperen S. Bingoel, Frederik Schlottmann, Clarissa Plinke, Khaled Dastagir, Doha Obed, Anieto Enechukwu, Thorben Dieck, Lukas Wellkamp, Jasmin Sarah Hanke, Christian Kühn, Arjang Ruhparwar, Nicco Krezdorn, Peter M. Vogt

**Affiliations:** Department of Plastic, Aesthetic, Hand and Reconstructive Surgery, Burn Center, Hannover Medical School, Hannover, Germany; Division of Plastic Surgery, Department of Trauma, Orthopedic and Plastic Surgery, 9177 University Medical Center Göttingen, Göttingen, Germany; Department of Cardiothoracic Surgery, Hannover Medical School, Hannover, Germany; Department for Plastic and Breast Surgery, Sjaelland University Hospital, Roskilde, Denmark

**Keywords:** vv-ECMO, acute respiratory distress syndrome, acute inhalation injury, burn surgery, extracorporeal membrane oxygenation, burns

## Abstract

**Objectives:**

The treatment of acute respiratory distress syndrome (ARDS) in burn patients remains a major challenge. Veno-venous extracorporeal membrane oxygenation (vv-ECMO) is a standard treatment for severe ARDS today. But reports on survival outcome in burn patients remain variable in the literature. The aim of this study is to identify factors that may influence survival and therapy outcomes in this distinct patient population.

**Methods:**

A single-center retrospective study was conducted in the burn intensive care unit (BICU). Inclusion criteria were the use of vv-ECMO for ARDS after burn injuries. The data analyzed included general medical data and various parameters from the BICU.

**Results:**

Between January 2012 and December 2022, 21 consecutive adult patients were identified who underwent vv-ECMO treatment. Five patients (24 %) survived the therapy and could be discharged, and 16 patients (76 %) succumbed to their disease. A higher TBSA affected, lower pH in arterial blood gas analysis after 24 and 36 h, multiorgan dysfunction syndrome (MODS), renal insufficiency, and renal replacement therapy were significantly associated with a lethal outcome.

**Conclusions:**

The data from the present study showed an overall mortality rate of 76 %, which is unsatisfactory compared to the literature. This could be explained by complicating factors such as MODS, renal failure, and renal replacement therapy. However, the indication for vv-ECMO must be adapted to the individual situation of the respective patient. Due to the additional higher risk for complications, the utilization of ECMO therapy should be reserved for specialized burn centers with an interdisciplinary setting.

## Introduction

The treatment of acute respiratory distress syndrome (ARDS) in burn patients remains a major challenge and has a significant influence on the clinical course. In addition to lung-protective ventilation and other conservative measures like prone positioning, invasive treatment modalities, e.g., veno-venous extracorporeal membrane oxygenation (vv-ECMO), are a standard treatment for severe ARDS today [1], 2].

Burn patients with moderate to high affection of body surface area frequently present with ARDS. Approximately 10 % of all intensive care unit (ICU) patients meet the criteria for ARDS, while the incidence amounts to 28–49 % in burn patients, respectively [3], [4], [5], [6].

Severe burns result in tissue damage, which can lead to systemic inflammatory response syndrome (SIRS) with multiorgan dysfunction syndrome (MODS) due to leukocyte activation and the production and release of proinflammatory mediators [7].

Since the CESAR trial in 2009, the use of ECMO in the treatment of ARDS has become widespread worldwide and pushed the frontiers of intensive care medicine [8]. Burn care has followed this trend in the last decade, which is documented by numerous publications. However, the procedure remains controversial in the case of burns due to the pronounced risk profile.

While overall survival rates after ARDS in nonburn patient populations are comparatively high [9], 10], reports on survival outcome in burn patients remain variable in the literature. Various studies lead to conflicting conclusions regarding mortality, risk, and the justifying indication for vv-ECMO [11], [12], [13], [14], [15].

Yet, there is no evidence for specific factors that might have an impact on mortality during therapy. Furthermore, none of the international burn guidelines offer specific recommendations with regard to vv-ECMO application in cases of severe respiratory insufficiency in this distinct patient population [16], 17]. The aim of this study is to identify factors that show an association with survival and death following vv-ECMO therapy in order to optimize decision making for vv-ECMO application in burn care.

## Materials and methods

### Patient selection and data collection

This single-center retrospective study was conducted in the burn intensive care unit (BICU) of Hannover Medical School, (Hannover, Germany). With six beds, the burn intensive care unit covers a major area in northern Germany and is also the only burn unit in Lower Saxony, providing burn intensive care for over eight million people. Inclusion criteria were the use of vv-ECMO, burn trauma, or acute inhalation injury. Patients with venoarterial-ECMO-therapy (va-ECMO) or absence of thermal injuries were excluded.

Epidemiologic, surgical, and medical data from the hospital information systems (SAP, Walldorf, Germany; m.life, medisite, Hannover, Germany) were collected.

Analyzed data comprised of age, sex, weight, height, body mass index (BMI), smoking status, total body surface area (TBSA), abbreviated burn severity index (ABSI) according to Tobiasen [18], acute lung injury (ALI), surgical therapy, settings of the respirator, blood gas analysis, comorbidities, sepsis-related organ failure assessment scores (SOFA-score), prone position, complications, and the occurrence of septic shock as well as MODS. All parameters were collected in a 12 h interval if applicable, respectively. Regarding vv-ECMO application, the cannulation technique as well as the cannulas used were noted. The setup was provided by the Department of Cardiothoracic Surgery. The data in this study were collected entirely at Hannover Medical School.

The study was approved by the institutional ethical board of Hannover Medical School (ethics approval number 8764_BO_K_2019, date of approval: 11/25/2019) and was in line with the ethical standards of the Declaration of Helsinki.

### Statistical analysis

Statistical analysis was performed using GraphPad Prism (Version 9.3.1 (350) for MacOS Monterey, GraphPad Software, San Diego, CA, USA). For continuous variables, the data are displayed as mean and standard deviation. For categorical variables, frequencies with percentage distribution are presented.

In terms of comparison of the data, Fisher’s exact test was performed for categorical variables, and Mann–Whitney U test was used for continuous variables. The tests were two-sided. Statistical significance was determined as p ≤0.05. Odds ratio was calculated for the categorical variables.

## Results

Between January 2012 and December 2022, 21 consecutive adult patients were identified who underwent vv-ECMO treatment for acute lung injury or ARDS associated with severe thermal injuries. Two patients were excluded due to nonthermal injury (postinfectious purpura fulminans) and due to the application of va-ECMO for cardiac failure. All baseline parameters are displayed in Table 1.

**Table 1:** Patient demographics (n=21).

Variable	Total (n=21)	Nonsurvivor (n=16)	Survivor (n=5)	p-Value
Age (mean)	47.0±14.05	47.50±14.45	45.40±14.12	0.5579
TBSA, %	36.43±24.43	41.75±24.45	19.40±16.32	0.0524
ABSI (mean)	8.524±2.713	9.125±2.527	6.600±2.608	0.1032
BMI, kg/m^2^	27.92±7.599	26.94±5.771	31.8±12.15	0.5609
SOFA-score pre-ECMO (mean)	10.67±3.498	11.4±3.405	8.200±2.775	0.1035
Initial COHb (mean)	8.348±7.310	7.619±7.157	10.68±8.136	0.1998
Gender	21 (100 %)	16 (100 %)	5 (100 %)	>0.9999
Female	5 (23.8 %)	4 (25 %)	4 (80 %)	
Male	16 (76.2 %)	12 (75 %)	1 (20 %)	
Acute lung injury	16 (76 %)	13 (81.2 %)	3 (60 %)	0.5528
Nicotine	15 (71 %)	12 (75 %)	3 (60 %)	0.5975
Alcohol abuse	7 (33.3 %)	6 (37.5 %)	1 (20 %)	0.6244
Septicemia	6 (28.5 %)	6 (37.5 %)	0	0.2621
Renal failure	15 (71.4 %)	15 (93.8 %)	0	0.0003
Renal replacement therapy	11 (52.3 %)	11 (68.8 %)	0	0.0124
MODS	13 (61.9 %)	13 (81.2 %)	0	0.0028
Cardiac disease	6 (28.5 %)	5 (31.3 %)	1 (20 %)	>0.9999
Pulmonary disease	2 (9.5 %)	2 (12.5 %)	0	>0.9999
Pneumonia	9 (42.9 %)	7 (43.8 %)	2 (40 %)	>0.9999
Prone belly	16 (76.1 %)	11 (68.8 %)	5 (100 %)	0.2776
pH-level pre-ECMO (mean)	7.276±0.1239	7.258±0.1047	7.334±0.1733	0.4357
Horovitz pre-ECMO (mean)	134.9±115.6	140.6±129.4	116.6±59.04	0.4819

The individual parameters are shown as mean values with standard deviation and as absolute numbers with percentage distribution. Mann–Whitney U test was used for analysis (TBSA, total body surface area; ABSI, abbreviated burn severity index; BMI, body mass index; MODS, multiorgan dysfunction syndrome).

**Figure 1:**
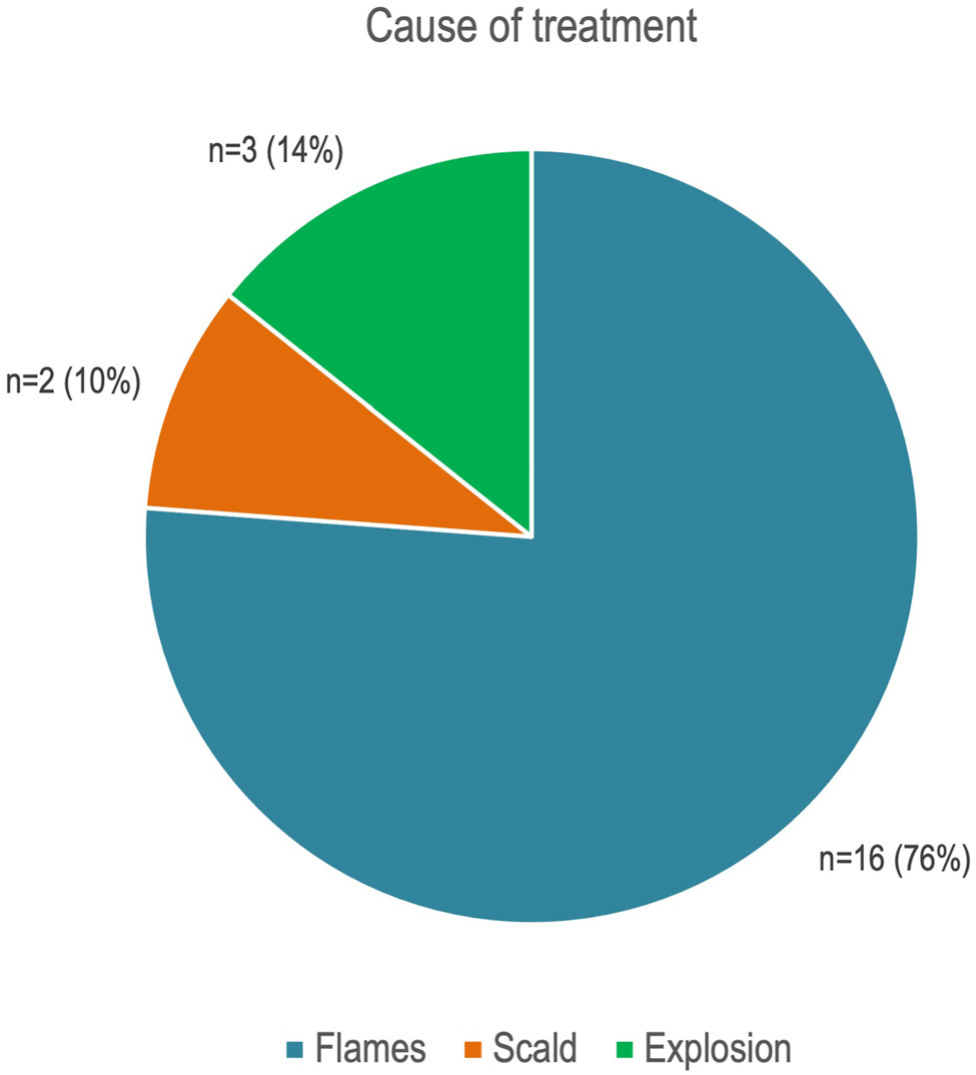
The main reason for hospitalization and vv-ECMO therapy in 76 % of cases was flame burns, followed by explosion (14 %) and scalding (10 %).

Within the study group of 21 patients, the mean age was 47.00±14.05 years, (range 16–65 years), and 76 % were male. The overall survival rate was 24 % (five patients). The TBSA affected was 36.43±24.43 % (range 0–85 %). The burn injury etiology could be stratified into flames (76 %, 16 patients), explosion (14 %, three patients), and scald (10 %, two patients) (Figure 1).

The mean BMI was 27.92±7.599 kg/m^2^ (range 18–52.5 kg/m^2^). Fifteen patients were active smokers (71 %). Sixteen patients (76 %) had ACL due to the initial thermal injury; five patients did not have signs of ALI. The indication for vv-ECMO was ARDS in all patients. The mean ABSI was 8.524±2.713, (range 4–14). The mean SOFA-score before vv-ECMO was 10.67±3.498, (range 5–19).

Mean length of ECMO time was 5,540±5,505 min (range 614–21,047 min).

Mean Horovitz index before the start of ECMO therapy was 134.9±115.6 (range 44–464).

Sixteen patients (76 %) had to be in prone position as part of the therapy during vv-ECMO.

Five patients (24 %) survived the therapy and could be discharged, and 16 patients (76 %) succumbed to their disease. In one patient who had postmortem forensic autopsy, hyperinflation of the lungs and substantial soot deposits were seen.

In 20 patients, the afferent cannula was a NovaPort^®^ cannula (Novalung^®^, Fresenius Medical Care, Heilbronn, Germany) with a size of 13, 15, 17, or 19 French (Fr.), and the efferent cannula was a HLS cannula (Maquet, Getinge Group, Göteborg, Sweden) with a size of 21, 23, or 25 Fr. In one patient, Edwards Lifesciences cannulas (Edwards Lifesciences Services, Irvine, CA, USA) with a size of 20 Fr. were used. Ultrasound guidance and percutaneous Seldingers’ technique were used for cannulation.

**Figure 2:**
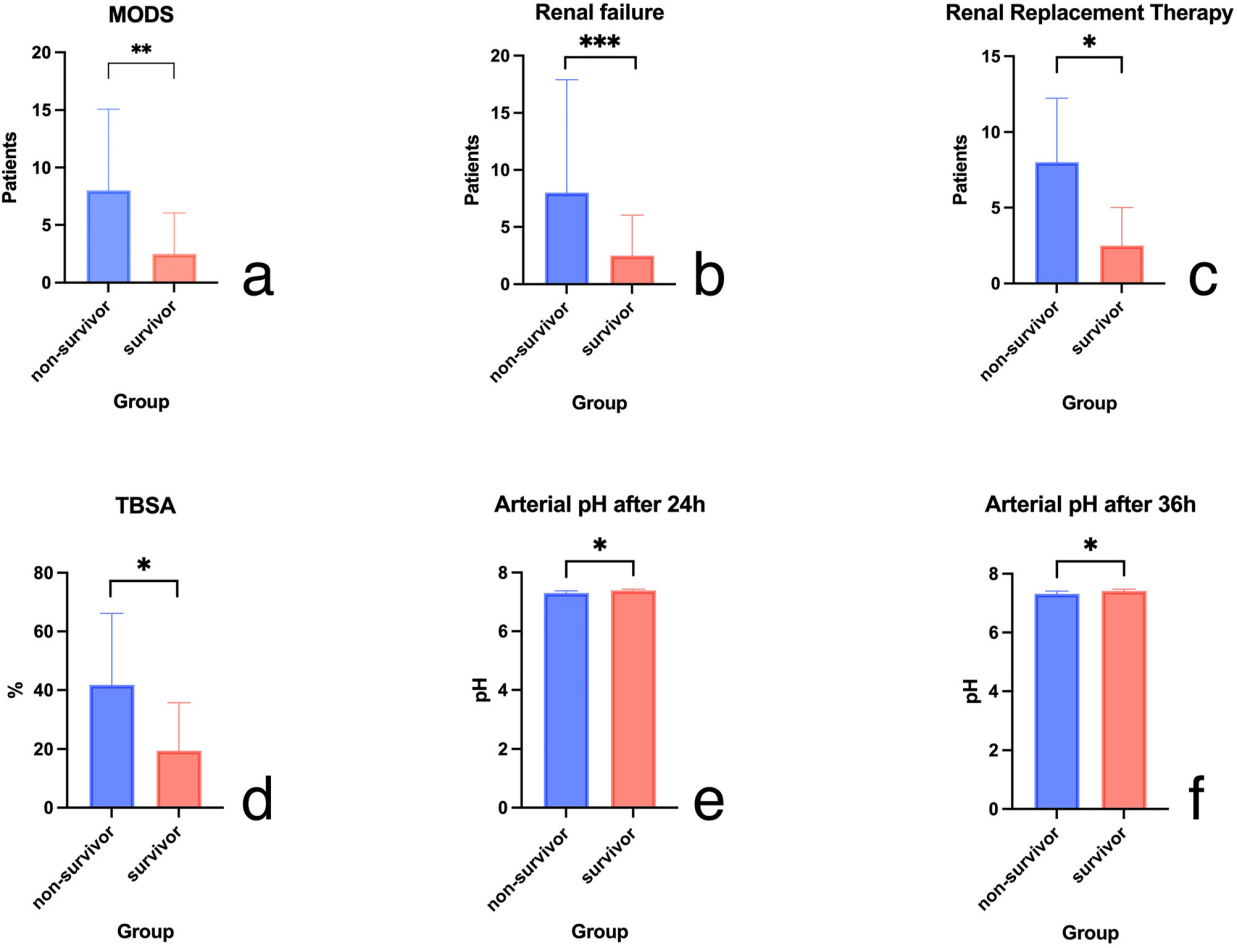
Statistically significant parameters with impact on vv-ECMO-therapy. Panel (a) shows the difference of multi organ dysfunction syndrome (MODS) between the two groups with a p=0.0028. Panels (b) and (c) show the difference for renal failure and renal replacement therapy with p=0.0003 and p=0.0124, respectively. The total body surface area affected (d) was significantly different between both groups with p ≤0.05. Panels (e) and (f) show the significance for the measured arterial pH values at 24 and 36 h post vv-ECMO implantation with p=0.0281 and p=0.0363, respectively. The other examined parameters were not significant.

Two groups were established for the statistical tests (nonsurvivors vs. survivors). The following parameters were significantly associated with death when comparing both groups following vv-ECMO therapy: higher TBSA (45.5 vs. 15 %; p=0.05) affected, lower pH in arterial blood gas analysis after 24 h (7.300 vs. 7.380; p=0.02) and 36 h (7.315 vs. 7.430; p=0.03). Multiorgan dysfunction syndrome (13 patients vs. 0 patients; p=0.0028), renal insufficiency (15 patients vs. 0 patients; p=0.0003), and renal replacement therapy (11 patients vs. 0 patients; p=0.0124) were also found to be statistically significant variables (Figure 2).

The following parameters showed a trend albeit without statistical significance: ABSI score upon admission (9 vs. 6; p=0.10), SOFA-score before vv-ECMO implantation (8 vs. 10; p=0.10), and Horovitz index 36 h post vv-ECMO implantation (136.5 vs. 207; p=0.07). All other parameters showed no statistical significance.

Six patients (28.5 %) had complications associated with vv-ECMO. Direct complications included diffuse bleeding with clotting of the vv-ECMO in one patient, as well as blood circulation insufficiency of the lower leg in one patient. The following were rated as indirect complications: gastric bleeding in one patient, disseminated intravascular coagulation (DIC) in two patients, and acquired severe coagulation disorder in one patient.

**Figure 3:**
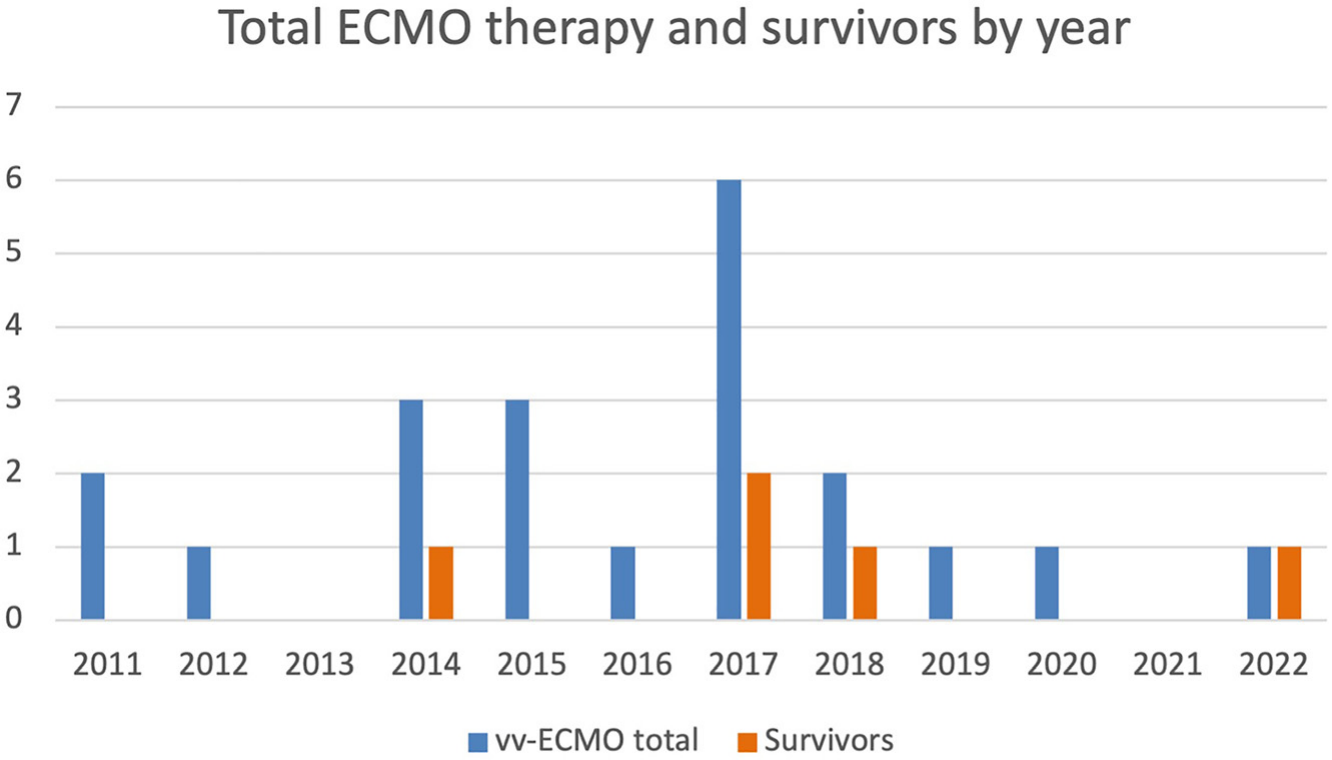
The annual distribution of ECMO cases in our burn unit shows 1–3 cases per year from 2011 to 2016 with only one patient surviving. Cases peaked in 2017 with six patients, of whom two survived. After that, the number of cases decreases again significantly with 1–2 cases per year and only two surviving in total.

The distribution of ECMO cases over the past 12 years is shown in Figure 3. Most cases were observed in 2017 with six cases in total and two surviving patients. Odds ratios for the categorical variables can be found in Table 2.

**Table 2:** Fisher’s exact test and odds ratio for categorical variables.

Variable	OR	95 %-CI	p-Value
Gender	0.75	0,05005–9,767	>0.99
Acute lung injury	2.889	03,606–19,20	0.5528
Comorbidities	2.889	03,606–19,20	0.5528
Cardiac diseases	1.818	01,645–26,21	>0.99
Pulmonary diseases	Infinity	01,401–∞	>0.99
Alcohol abuse	2.4	02,340–33,68	0.6244
Nicotine	2	02,679–12,60	0.5975
Prone belly	0	0.000–2,718	0.2776
MODS	Infinity	3,179–∞	0.0028
Renal failure	Infinity	6,078–∞	0.0003
Renal replacement therapy	Infinity	1,902–∞	0.0124
Pneumonia	1.167	01,916–7,924	>0.99
Septicemia	Infinity	0.5232–∞	0.2621

## Discussion

We hereby present a retrospective cohort study with 21 patients who received vv-ECMO therapy for ARDS after thermal injuries that resulted in respiratory failure. The major findings were that MODS, renal failure, and renal replacement therapy were associated with a significantly higher mortality rate during vv-ECMO therapy. As expected, the data showed that a higher TBSA as well as a lower pH value in arterial blood gas analysis after 24 and 36 h post vv-ECMO implantation were associated with significantly higher mortality rates. A trend toward higher mortality was seen in patients with an increased ABSI, SOFA-score, and reduced Horowitz index after 36 h following vv-ECMO implantation.

The use of vv-ECMO in general is a significant advancement in the field of intensive care treatment for critically ill patients [19]. It quickly advanced to a crucial component in the treatment for pulmonary insufficiency, which is primarily brought on by ARDS. Unfortunately, the clinical outcome remains controversial [20].

ARDS in burn patients is one of the major complications with mortality rates of up to 31 % [21]. The therapeutic value of vv-ECMO in burn care is not well understood but has nonetheless gained momentum over the past decades.

Furthermore, it seems that the therapeutic concept of vv-ECMO cannot simply be transferred to the distinct population of burn patients. This cohort is vulnerable to burn inflammation-related consequences; hence, a careful examination of potential medical consequences following ECMO application is necessary.

In general, the complications can be divided into ECMO-related and nonrelated. ECMO-related complications include pump or oxygenator dysfunction, thrombosis, and cannula dislocation, while other physiological complications consist of infections, hemolysis, bleeding, or SIRS following ECMO itself [22], 23]. These should not be underestimated in burn patients since the burn disease itself can cause fulminant SIRS marked by severe imbalance of cytokines and the coagulation system [24].

The need for anticoagulation during ECMO therapy can provoke hemorrhage. The burn injury itself can cause coagulopathies with resulting imbalances of coagulation factors as part of the entire inflammatory process. The danger of bleeding is particularly significant in burn patients since early and aggressive surgical therapy is necessary to lessen the necrotic burden [25]. Both factors can potentiate a progressive coagulopathy and lead to an even more difficult clinical course and life-altering outcomes [24].

In 2016, Soussi et al. reported high mortality rates in burn patient treated with vv-ECMO (90-day survival of 28 %) and did not consider the therapy to be advisable until better quality data were registered [11]. However, the problem of a systematic approach to ECMO therapy in burn patients currently remains. A comprehensive, and in the ideal case, randomized, prospective study is frequently challenging to achieve because of the relatively small number of patients. The available data are, therefore, in most cases of retrospective nature.

Another problem with the low number of cases is due to the epidemiology of burns and their global distribution. In a recent epidemiological study on burns, Yakupu et al. showed that there is a global trend toward an increase in the number of new burn cases [26]. But the total incidence of burn injuries is decreasing in high-income nations compared to low-income countries [27]. Nevertheless, vv-ECMO is likely not available in low-income countries due to the significant structural and technological effort, which also makes systematic studies more difficult. According to a web-based survey, even in high-income nations like Canada and the United States, approximately only half of the burn centers have used ECMO in burn patients [17].

In 2017, Burke et al. described a heterogeneous group of 58 patients (76 % vv-ECMO) [28]. The data from this study show, in line with the present data, that renal insufficiency and renal replacement therapy are associated with an increase in the risk of mortality. Interestingly, it is also suggested that patients with high TBSA affected present with reduced survival probabilities. The data from the current study confirm this assumption, as in the present patient cohort a significantly higher mortality rate was found for patients with a higher TBSA affected.

Song et al. highlighted that both the pulmonary and renal system are sensitive organ systems, which play a key role in patients with MODS [23]. In their review published in 2021, they reported a combination therapy with ECMO and continuous renal replacement therapy (CRRT).

Based on previously published data, they recorded survival rates after given combination therapy of 50–100 %, however, without detailed patient characteristics. In addition, they treated three patients who died due to MODS from wound infections. They emphasize that the timing of the combination therapy on the surgical treatment of extensive wounds remains challenging. However, our data indicate that patients with renal insufficiency and dialysis have a significantly poorer outcome in terms of survival. Our study is also at odds with an earlier study conducted by Szentgyorgyi et al. who could show in five patients that ECMO and renal replacement therapy can be combined successfully [12].

A systematic review by Folkestad et al. showed that the renal system is very sensitive in the context of thermal injuries and that the presence of acute kidney injury (AKI) was related to an increase of length of stay (LOS) and mortality [29]. They determined that the incidence for AKI was between 30 and 46 % in burn patients treated within an ICU setting. Risk factors also included high TBSA, a high ABSI and SOFA score, as well as the presence of inhalation trauma, which approximates our data.

To be able to compare mortality rates between different studies, the parameters of the specific studies must be analyzed in detail. For example, Eldredge et al. achieved a survival rate of 87.5 % in their study [30]. From our point of view, a comparison with the data from the current study is not expedient, since the pediatric patient proportion is significantly higher (eight patients in total; two adults and six pediatric patients). Nevertheless, they also emphasize that correct patient selection and early decision making are crucial for survival.

Ainsworth et al. published a case series with a patient cohort of 14 patients [31]. Looking only at the burn patients (n=11), the mortality rate for this cohort was 54 %. According to the authors, this was the lowest mortality rate that had been published up to that point of time. Comparing parameters such as ALI and the TBSA, it is noticeable that in our cohort the mean rate of ALI was 76 % compared to 33 %, respectively. Furthermore, the mean TBSA is 36 % in the current study vs. 27 %, respectively. Therefore, a possible explanation for the significantly higher mortality rate of 76 % in the current study can be a more severely ill patient cohort.

Dadras et al. were able to achieve a low mortality rate of 37.5 % in eight patients with almost the same TBSA (36 vs. 37 %) over a time period of 2 years [32].

Their assumption appears reasonable that many cases in a short period of time lead to an improved learning curve that can enhance the outcome. In contrast, our cases are spread over 12 years. The distribution from 2019 in our cohort showed only one case per year (Figure 3). One possible explanation for this could be, in addition to the lower number of burn cases mentioned above, that due to the previous experience of the practitioners, vv-ECMO was indicated more cautiously.

A work by Fouché et al. recently showed that ECMO is a feasible treatment option and can lead to relatively good survival rates [33]. In a group of patients comparable to the present study (mean TBSA 37 %, inhalation injury in 83 % of the patients), a survival rate of 77.7 % following vv-ECMO was presented.

However, two systematic reviews only 9 years apart (2013 vs. 2022) offer hardly any new insights. In 2013, Asmussen et al. concluded that “the overall data suggest that there is no improvement in survival for burn patients suffering acute hypoxemic respiratory failure, with the use of ECMO” [34]. As noted by the authors, the number of patients was insufficient, and the level of evidence was low due to the retrospective nature of the studies.

In 2022, Chiu et al. had more comprehensive data, which they statistically analyzed. Contrary to other expectations, their calculations delineated “that extracorporeal membrane oxygenation recipients have significantly higher mortality rates compared to their predicted mortality rates calculated using their revised Baux scores” [35]. Based on their data, they disagreed with the routine use of ECMO in burn patients. The only subgroup that benefited from vv-ECMO was patients with inhalation injury, major burns, and a revised Baux scores exceeding 90 points. The pooled mortality rate in the vv-ECMO subgroup was 41.8 %. One of the main conclusions of the study was the early consideration of transferring these patients to an ECMO center.

A critical factor in the initiation of vv-ECMO therapy is timing. Statistical analysis revealed no significant difference between the two groups in terms of time duration from admission to start of vv-ECMO (nonsurvivors vs. survivors: 10,247 vs. 6,799 min; p=0.5476). However, in everyday clinical practice, we have tended to initiate vv-ECMO therapy earlier. This may have a difference in the clinical outcome in terms of survival.

When determining the indication, several factors were taken into account during interdisciplinary rounds, including the presence of sepsis, the ventilation situation with high pressures, and the patient’s overall prognosis in the context of the burns. This indication was given individually, in the early years also as a last resort. With increasing experience, the indication was given more cautiously.

In summary, our research has highlighted that MODS, especially renal failure, and the need for dialysis, is associated with a significantly higher mortality rate. In addition, the data showed that a higher TBSA affected, and a lower pH value in arterial blood gas analysis after 24 and 36 h post vv-ECMO implantation has a significantly higher mortality.

Furthermore, the present results showed that patients with an increased ABSI, SOFA-score, and reduced Horovitz index after 36 h after vv-ECMO implantation showed a trend toward increased mortality.

Our results present with limitations. The first is the limited number of cases of 21 patients included. The second is the retrospective character of our study. Thus, the statistical results need to be interpreted with caution. The interpretation of the p-value in one direction and in the other can distort decisions in clinical practice.

Furthermore, the combination of numerous factors such as the influence of the continuous flow system of the ECMO, its proinflammatory characteristics on the immune system, the administration of blood products, and their impact on microcirculation and wound healing remain unclear [22].

## Conclusions

The data from the present study suggest that in vv-ECMO treated burn patients suffering from ARDS, renal insufficiency, MODS, higher TBSA affected, and low 24 h and 36 h pH values are associated with reduced survival probability. In our patient cohort, the overall mortality rate was 76 %, which is poor compared to the literature. Nonetheless, one must also understand that mortality is highly dependent on the severity of burns and comorbidities.

Although mortality rates vary widely across studies, it seems that there is sufficient evidence in the literature that encourages the use of vv-ECMO in ARDS in the context of thermal injuries in selected patients. However, the indication for vv-ECMO must be adapted to the individual situation of the respective patient.

In our opinion and clinical experience, the decision for ECMO therapy is always an individual and patient-related decision that must consider cardiac, pulmonary, and renal diseases, burn severity, and the overall prognosis. Early access to vv-ECMO therapy seems to be beneficial in distinct burn patients. Still, due to the additional high risk of complications, the utilization of ECMO therapy should be reserved for specialized burn centers with an interdisciplinary setting.
